# Near Visual Acuity Measurements by Community Screeners Using Digital (Peek) Testing Versus Conventional Charts in India

**DOI:** 10.1167/tvst.14.8.24

**Published:** 2025-08-19

**Authors:** Shalinder Sabherwal, Mohd Javed, Elanor Watts, Marzieh Katibeh, Alice Pintus, Nam Thaker, Sergio Latorre-Arteaga, Vince Hewitt, Darren Coverley, Nigel M. Bolster, Andrew Bastawrous

**Affiliations:** 1 Department of Community Ophthalmology and Public Health Research, Dr. Shroff's Charity Eye Hospital, New Delhi, India; 2 International Centre for Eye Health, London School of Hygiene and Tropical Medicine, London, UK; 3 Dr. Shroff's Charity Eye Hospital, New Delhi, India; 4 Peek Vision, Berkhamsted, UK; 5 Tennent Institute of Ophthalmology, Glasgow, UK; 6 Department of Ophthalmology, Rigshospitalet, Copenhagen University Hospital, Glostrup, Denmark; 7 Public Health Research Group, University of Alicante, Alicante, Spain

**Keywords:** visual acuity, visual acuity charts, presbyopia, epidemiology, near vision

## Abstract

**Purpose:**

The Peek digital near vision test has been previously validated in a trial setting; here it is assessed in clinic (stage 1) and community (stage 2) settings.

**Methods:**

The study was carried out in the catchment area of Dr. Shroff's Charity Eye Hospital, Mohammadi, Uttar Pradesh, India, with a total of 768 participants. Stage 1 assessed the interobserver variability of Peek near vision impairment (NVI) screening in 168 clinic participants, with three trained community screeners. Stage 2 compared Peek to conventional chart testing for NVI screening using Cohen's kappa coefficient, sensitivity, and specificity and, for quantitative near visual acuity (NVA) measurement, using Bland–Altman limits of agreement (LoA) in 600 participants with two screeners.

**Results:**

In stage 1, interobserver variability using Peek ranged from 96.43% to 98.21% (kappa = 0.92–0.96). In stage 2, there was overall agreement in 95.8% of cases (kappa = 0.91). Peek testing had a sensitivity and specificity of 91.25% (95% confidence interval [CI], 87.22–94.1) and 99.41% (95% CI, 97.86–99.84), respectively. For NVA testing, the 95% LoA between Peek and chart testing were within −0.11 and +0.07 logMAR. Mean test time was 40.3 seconds (95% CI, 38.8–41.7) for Peek versus 46.6 seconds (95% CI, 45.5–47.7) for a conventional chart.

**Conclusions:**

The previously demonstrated validity of Peek testing was maintained when used by trained community screeners.

**Translational Relevance:**

The Peek near vision test can be used as a validated method of NVA/NVI measurement in research, clinical and community settings.

## Introduction

The World Health Organization and the *International Classification of Diseases*, 11th Revision,^1^ define near vision impairment (NVI) as the inability to see N6 at 40 cm. An estimated 826 million people worldwide have near vision impairment due to uncorrected presbyopia,^2^ which could be easily managed with reading glasses. Near vision correction improves productivity,^2^^,^^3^ income,^4^ and quality of life,^5^^,^^6^ contributing to 12 of the United Nations sustainable development goals, and therefore is a priority of the World Health Organization (WHO) within their SPECS 2030 initiative. However, estimates of prevalence are made less reliable by a lack of standardization in near visual acuity (NVA) testing^7^ compared to distance visual acuity (DVA), contributing to wide variability in epidemiological estimates; alternative modeling has determined a global estimate of 510 million people with uncorrected presbyopia.^8^ NVA is less commonly tested and recorded than DVA and a wide range of methods are available to test NVA,^7^ including single optotype charts,^9^ and continuous text reading charts,^10^ with further variability in testing distance.^7^ Although there are advantages to each of these options, there is a need for standardization across programs and epidemiological studies to allow comparison and combination of data. There is no universally accepted gold-standard test for NVA. The WHO *Package of Eye Care Interventions*^11^ suggests use of Snellen charts with letters, pictures, numbers, and tumbling “E” charts for VA testing in the community, and, for secondary and tertiary care, any of the following: logMAR charts, LEA Symbols near vision cards, Sloan letters, HOTV charts, or Kay Pictures. Any standard test for global use must not be language specific or literacy dependent. A novel digital near vision test has been developed and validated by Peek Vision Ltd. (“Peek”), under strict trial conditions, in Lahan, Nepal.^12^ In this study, we assessed the use of the Peek near vision test in a clinical setting and community program setting, in Northern India.

## Methods

### Objectives

1. To assess sensitivity and specificity of NVI screening with comparison of interobserver variability (IOV) using Peek digital testing and conventional chart-based testing, carried out by trained community screeners and by optometrists (treated as gold standard).
2. To assess agreement of quantitative NVA testing using Peek and conventional charts using Bland–Altman limits of agreement (LoA).
3. To compare the time required for testing with the Peek and conventional charts.

### Study Design and Participants

This was a cross-sectional observational interobserver variability/validity study. The study setting was the catchment area of Dr. Shroff's Charity Eye Hospital in Mohammadi, Uttar Pradesh, in Northern India. Test development has been described previously.^12^ Sample size was calculated for sensitivity and specificity analysis as per Buderer,^13^ using NVI prevalence among people 35 years and older of 0.3 (based on previous programmatic data from household community eye screening programs in the country), expected sensitivity of 90% and specificity of 95% (per previous validation study data), and confidence level of 95%. For stage 1 (clinic IOV), a precision of 0.1 and expected dropout rate of 5% were used, producing a minimum sample size of 129 participants. For stage 2 (community validation), a higher precision level and expected dropout rate were applied (0.05 and 10%, respectively), producing a minimum sample size of 513 participants for this part of the study. Inclusion criteria were age ≥ 35 years, confirmed via age identification documents, and being willing to provide consent and participate in the study. Exclusion criteria were age < 35 years, declining to participate, or inability to provide informed consent.

### Training

The screeners recruited did not have any previous work experience or background in eye care or health care. Their training was carried out over a period of 2 weeks by clinical optometrists. In the first week, they were oriented to the need for eye-care programs and common eye conditions (including presbyopia) in different age groups. They were trained to carry out a basic torchlight examination to differentiate a normal eye from an eye with common eye conditions. This was followed by explaining the principles behind DVA testing and training them on capturing DVA using Peek Acuity (Peek Vision, Berkhamsted, UK), followed by NVA using standard near vision charts and Peek near vision test. They spent one day in the clinics to observe and practice torchlight examination and vision testing. Screeners’ DVA testing was assessed by the clinical optometrist for 20 patients. The next week was spent carrying out screening under supervision in the community, with detailed feedback.

### Testing Protocol and Data Collection

Stage 1 testing was carried out in testing stations in the outpatient department of Mohammadi Hospital in May 2024. Stations were separated with secure, independent vision tests without verbal or non-verbal data contamination between the examiners (i.e., both participants and testers were masked regarding results at the other stations). Stage 2 testing was carried out as field testing within community eye care programs in the Mohammadi Hospital catchment area from July to August 2024 (see Fig. 3).

Monocular uncorrected distance vision in right and left eyes was measured using Peek Acuity and the results were inputted into an EpiCollect5 form (Centre for Genomic Pathogen Surveillance, Oxford, UK). Near vision was tested binocularly at 40 cm, measured by a precut cord, at a natural reading angle. Participants were randomized to start with either Peek testing or Tumbling “E” Near Point Vision Chart (Precision Vision, Woodstock, IL). Near vision was tested by one optometrist using the standard chart (gold standard) and via both modalities—Peek digital testing and Tumbling “E” charts—by three non-optometrist trained community screeners in stage 1, with the order randomized.

In stage 2, two screeners who already achieved good agreement with the gold standard team (optometrist) performed NV tests in the field by both modalities. The team consisted of two screeners to perform NV tests, a third screener to capture the timing of the test using the NV chart, and one study coordinator to manage the screening and also capture time taken with Peek. The study coordinator and one of the screeners visited each house.

Eligibility to take part was confirmed and consent obtained following explanation of the objectives of the study. Participants’ demographics were collected, including age, gender, and glasses ownership status. The screener then performed the near vision test using Peek and the coordinator recorded the time taken. The second screener then tested near vision using Tumbling “E” chart while the third screener captured the time taken. Screener order and role alternated. Near vision was tested both as a binary variable (NVI, inability to see N6 at 40 cm), and as a quantitative NVA measurement in both settings.

### Data Management

Data were collected using EpiCollect5 and a Peek Vision plugin app, including the Peek near vision test. Data were exported in csv and MS-excel format. Data cleaning was carried out using Excel (Microsoft Corporation, Redmond, WA), and statistical analysis was undertaken using Stata 16 (StataCorp, College Station, TX). Bland–Altman plots were created using R 4.2.0 (R Foundation for Statistical Computing, Vienna, Austria). Data and all appropriate documentation will be stored for a minimum of 5 years after completion of the study, including the follow-up period.

### Data Analysis

VA measurements were converted to logMAR for analysis. Treating the conventional Tumbling “E” test as a reference standard, the NVI screening function of the Peek test was assessed using Cohen's kappa coefficient, sensitivity, and specificity. The repeatability of screening results by Peek and conventional chart was reported by crude agreement percentage and Cohen's kappa, accompanied by 95% confidence intervals (CIs). The Bland–Altman LoA technique was used to evaluate the test–retest repeatability of quantitative NVA by the Peek test and the conventional Tumbling “E” test and to assess agreement between the Peek NVA testing with conventional Tumbling “E” testing. The differences in time taken between the conventional test and the Peek test are presented as box-and-whisker plots that display the median and interquartile ranges. The statistical differences in means of NV test times for the two modalities were also analyzed using a paired *t*-test.

### Ethical Considerations

The study was approved by the Institutional Review Board of Dr. Shroff's Charity Eye Hospital (reference no. IRB/2024/MAY/02). All participants gave free and informed written or thumbprint consent to participate. The study was conducted in accordance with the tenets of the Declaration of Helsinki.

## Results

### Participants

The clinic IOV sample was 168 participants, and the final community field testing sample was 600 participants, with a mean age of 50.6 ± 11.1 years; 53.0% were female. Participant characteristics are summarized in more detail in Table 1.

**Table 1.** Participant Characteristics for Stage 1 and Stage 2

Characteristics	Stage 1. Clinic IOV	Stage 2. Community
Gender, *n* (%)		
Female	86 (51.2)	318 (53.0)
Male	82 (48.8)	282 (47.0)
Age (y), mean ± SD (range)	49.1 ± 11.5 (35–82)	50.6 ± 11.1 (35–92)
Glasses worn for near vision, *n* (%)		
Bifocal or multifocal	20 (11.9)	50 (8.3)
Near vision/reading glasses	4 (2.4)	6 (1.0)
None	144 (85.7)	544 (90.7)
NV test result using standard chart, *n* (%)		
Pass	92 (54.7)	377 (56.2)
Fail	76 (45.3)	263 (43.8)
Total	168 (100)	600 (100)

### Stage 1. Clinic IOV

####  

Interobserver variability is shown in Table 2. The screeners achieved acceptable agreement in NV tests with the experienced optometrist using the standard chart. The comparison of IOV between screeners (with either chart or app) were also high in all comparisons, with kappa values of 0.96 to 0.98 for conventional chart testing between screeners, and 0.92 to 0.96 for Peek between screeners.

**Table 2.** Interobserver Variability for NVI Screening

Comparison	Agreement	Kappa	Standard Error
Conventional chart testing, screener and optometrist IOV			
Screener 1 vs. optometrist	85.71%	0.71	0.08
Screener 2 vs. optometrist	85.71%	0.71	0.08
Screener 3 vs. optometrist	87.50%	0.75	0.08
Conventional chart testing, IOV between screeners			
Screener 1 vs. screener 2	98.81%	0.98	0.08
Screener 1 vs. screener 3	98.21%	0.96	0.08
Screener 2 vs. screener 3	98.21%	0.96	0.08
Peek testing, IOV between screeners			
Screener 1 vs. screener 2	96.43%	0.92	0.08
Screener 1 vs. screener 3	98.21%	0.96	0.08
Screener 2 vs. screener 3	97.02%	0.94	0.08

### Stage 2. Community Validation

#### Binary NVI Screening

In the comparison of Peek and conventional chart testing for the presence or absence of NVI by different screeners, there was overall agreement in 95.8% of cases, with a kappa of 0.91. In general, 240 people failed testing with both modalities, and 335 passed with both. Where there was disagreement, there were two cases of passing with chart testing and failing with Peek testing and 23 cases of failing with chart testing and passing with Peek. Compared to testing with the chart, Peek testing had sensitivity of 91.25% (95% CI, 87.22–94.10) and specificity of 99.41% (95% CI, 97.86–99.84). Table 3 provides more details regarding NV screening accuracy by the Peek test in comparison with the standard chart.

**Table 3.** NVI Screening Test Evaluation in the Field

		Confidence Interval	
Parameter	Estimate (%)	Lower	Upper
Sensitivity	91.25	87.22	94.10
Specificity	99.41	97.86	99.84
Positive predictive value	99.17	97.04	99.77
Negative predictive value	93.58	90.54	95.68
Diagnostic accuracy	95.83	93.92	97.16
Cohen's kappa	0.91	0.83	0.99

#### Quantitative NVA Testing

**Figure 1.**
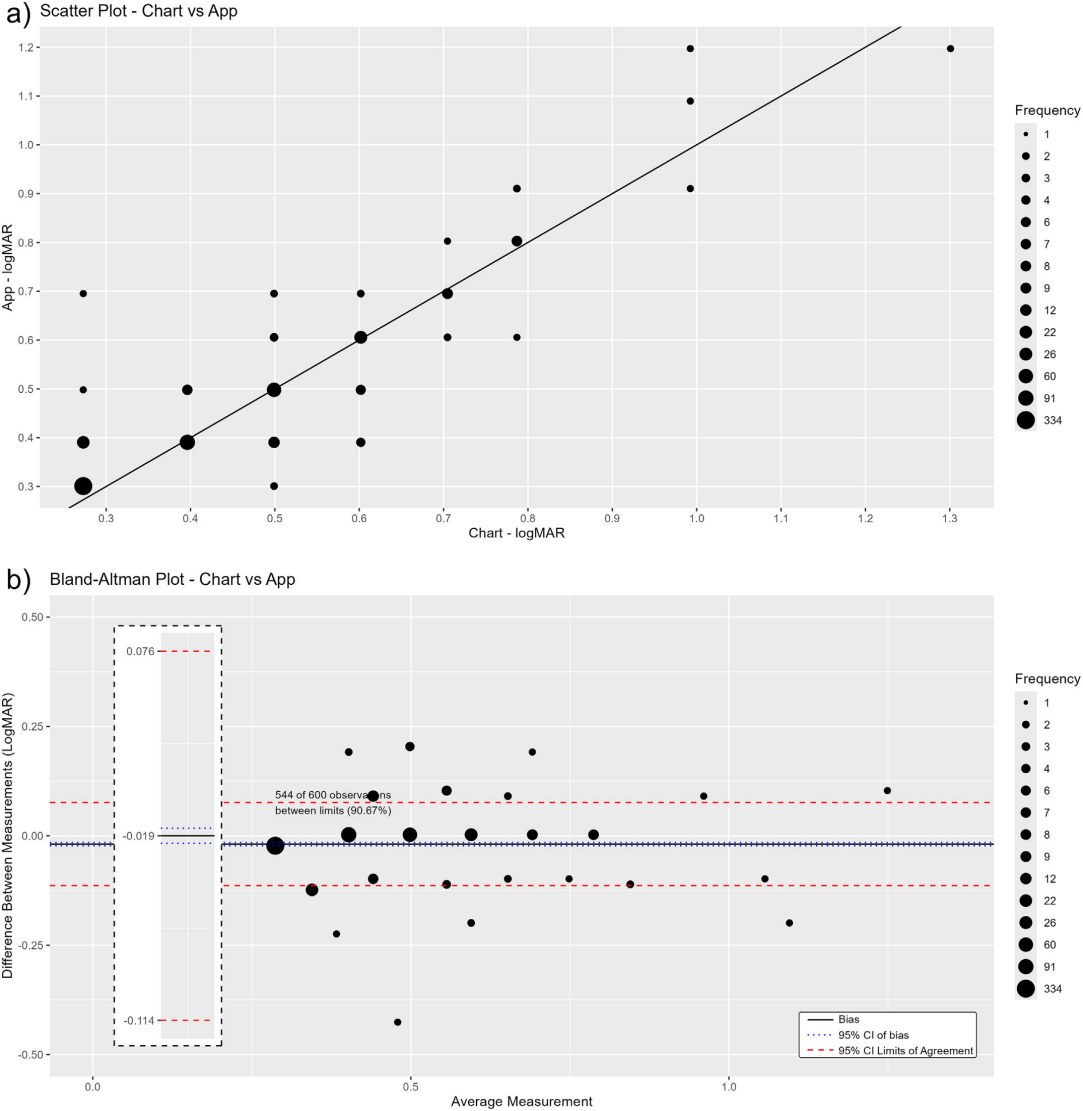
(**a**, **b**) Agreement in quantitative NVA testing between the Peek near vision test and conventional chart test by different screeners shown as a scatterplot (**a**) and as Bland–Altman LoA (**b**).

Agreement between Peek and chart testing by different screeners is depicted in a scatterplot and Bland–Altman graph in Figure 1. Bias was very low at −0.019 logMAR, and the 95% LoA between Peek testing and chart testing were within −0.11 and +0.07 logMAR.

#### Timing of Tests

**Figure 2.**
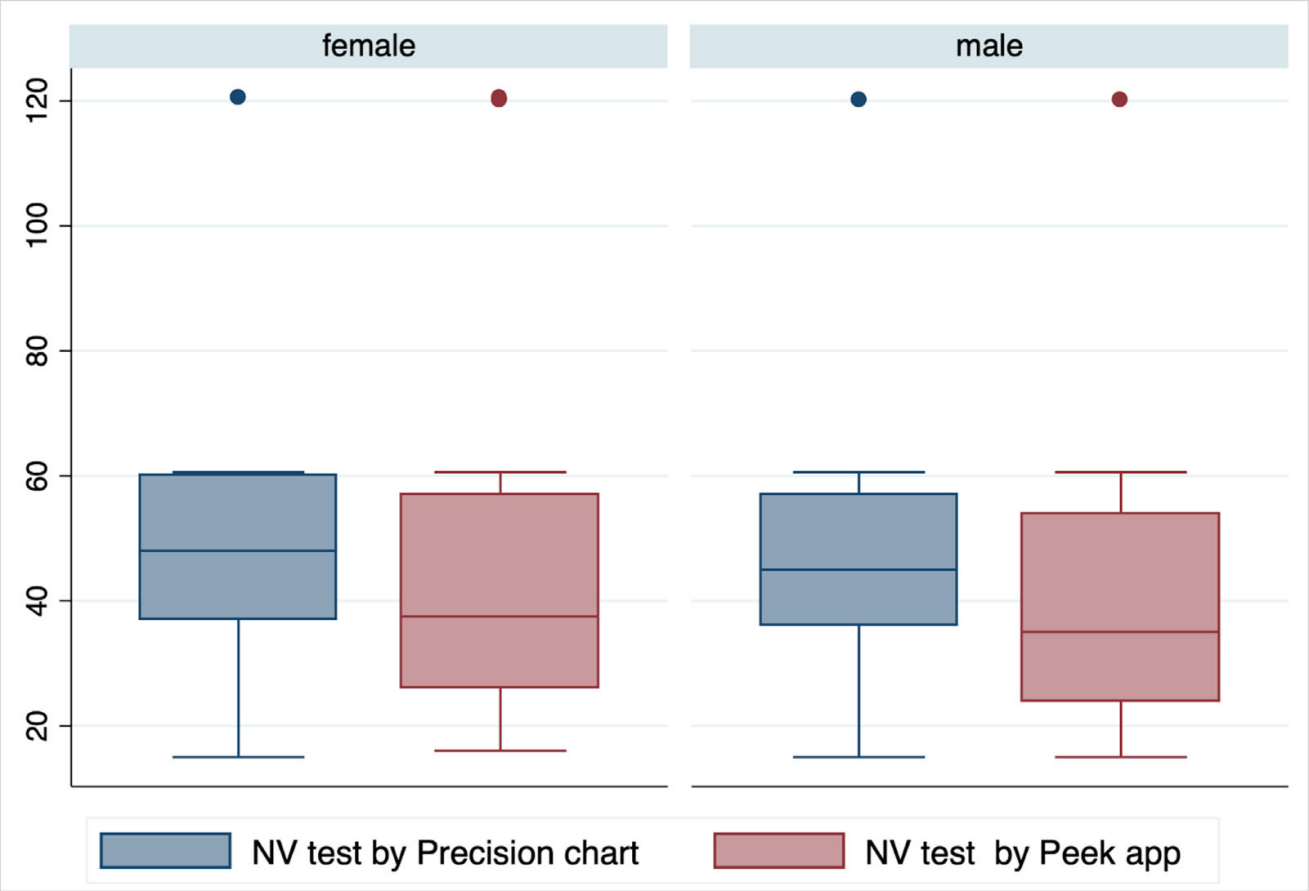
Box plot of time taken for NVA testing (seconds), with the Peek near vision test and conventional Precision Vision chart, by gender.

A comparison of the full test times (quantitative NVA) is shown in Figure 2^3^. The mean test time was faster for Peek (40.3 seconds; 95% CI: 38.8, 41.7) compared to the chart (46.6 seconds; 95% CI, 45.5–47.7; *P* < 0.001).

**Figure 3.**
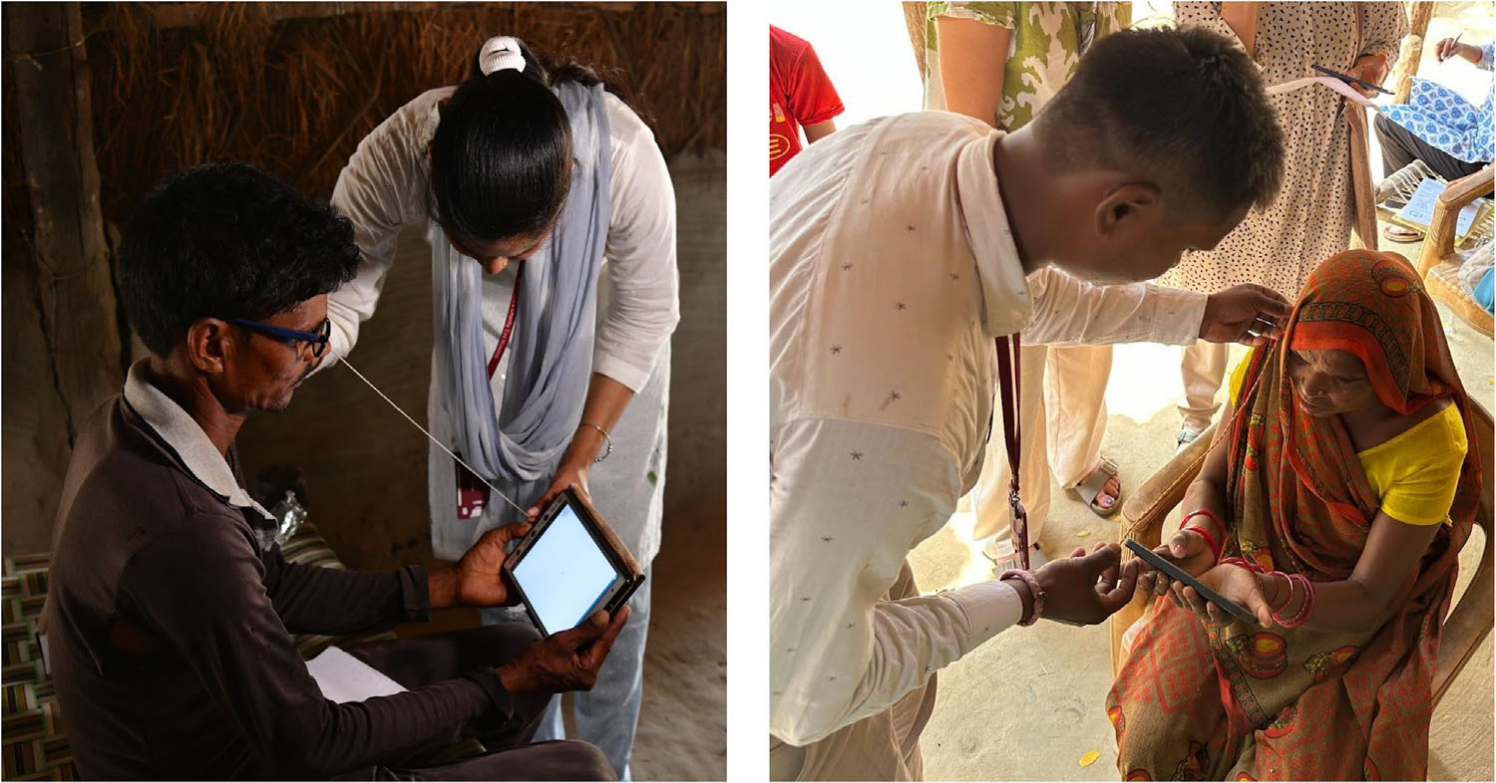
(**a**, **b**) Photographs of Peek testing in a community setting within stage 2 of the study. Photograph courtesy of Dr. Shroff's Charity Eye Hospital and Peek Vision.

## Discussion

The validity of the digital Peek near vision test in a strict trial environment has previously been demonstrated, with an overall agreement of 92.9% (kappa = 0.85) for NVI screening and 95% LoA between Peek and chart testing within −0.218 and 0.235 logMAR for NVA.^12^ Here, we assessed the repeatability and validity of Peek near vision test results in both a clinic setting and a community eye health program setting by non-optometrist screeners to reflect the environments for which the test is designed. The catchment population is representative of a rural setting with limited literacy (approximately 60%) in one of the most populous states in India.^14^

Near vision is known to be variable with prolonged accommodation,^15^ but, in spite of this, good interrater agreement was seen with use of the Peek near vision test in both settings, and the Peek test maintained good agreement with conventional chart testing. For NVI screening, overall agreement with the conventional chart was 95.8% (kappa = 0.91), higher than in the original validity study. For quantitative NVA, the 95% LoA between Peek and chart testing were within −0.11 and +0.07 logMAR. There was a bias of −0.019 logMAR, which is not clinically significant.

In a recent study with trial conditions comparing a different app-based NVA test (Eye Handbook) to a conventional test (Rosenbaum near vision card), Bland–Altman 95% LoA were −0.10 and +0.19 logMAR.^16^ Elsewhere, a comparison of the WHOeyes digital NVA test with Early Treatment Diabetic Retinopathy Study (ETDRS) near vision testing in a real-world setting found 95% LoA values ranging from −0.34 to +0.25.^17^ A similar study^18^ reported significant differences between NVA results with the SightBook mobile app and Rosenbaum near vision card (of 5.4 and 6.1 letters in right and left eyes, respectively), although acuities were repeatable within a modality. Another digital acuity test, OdySight, demonstrated 95% LoA of −9.75 and +10.82 letters compared to 40-cm Sloan ETDRS.^19^ As such, the Peek near vision test compares favorably to other available smartphone-based NV tests. Peek testing was also faster than with the conventional chart, with mean times of 40.3 and 46.6 seconds, respectively (*P* < 0.001). In large-scale community programs, this cumulative difference in time may allow for larger numbers of people to be screened.

Limitations of this study include lack of optometrist data using smartphone testing in stage 1. However, our initial IOV in this study demonstrated good interrater reliability between screeners with both chart and app. Another limitation is the small number of screeners in stage 2. Therefore, we must be cautious when generalizing the levels of agreement observed here, if used by different individuals or in a different regional context, as with any equivalent study design. However, these screeners’ background and experience did not differ significantly from those of the other >100 screeners active in the program. A potential limitation of the test itself is the requirement for access to a compatible smartphone, with sufficient screen pixel density; however, this barrier is being reduced, as smartphone penetration in India is now estimated to be up to 60%; over a billion Indians are thought to have smartphone internet access in 2025.^20^

The Peek near vision test can be applied in a wide range of ways, allowing further research. These include integrating the test within wider digital platforms, such as within community eye health or school eye health programs. The Peek near vision test is also suitable for use in research settings, and epidemiological survey. As an example, incorporation into the Rapid Assessment of Avoidable Blindness (RAAB7) software,^21^ will allow for calculation of near effective refractive error coverage. Development of an additional digital tool to support the provision of presbyopia correction (near vision glasses) would also be a useful future research topic.

In summary, the digital Peek near vision test was found to be reliable in clinic and community settings, as well as in previous trial condition validation.^12^ Presbyopia correction is being increasingly prioritized as a global development issue, and the improved standardization of near vision testing is a key step to reliably measuring and correcting NVI. To our knowledge, this is the first time a digital near vision test has been tested and validated directly at the household level by non-optometrist screeners and under real life conditions. This approach saves time for community healthcare workers, requires minimal training, and does not necessitate time for interpretation or recording of test results. All of these benefits can be enormously valuable in mass screening programs, where an extra minute saved during training or screening can mean identifying and helping one more person with near visual impairment—using a user-friendly diagnostic tool that enables timely, cost-effective intervention with near vision glasses.
